# Special Issue “Biotechnological Application of Photosynthetic Bacteria”

**DOI:** 10.3390/microorganisms11030619

**Published:** 2023-02-28

**Authors:** Hitoshi Miyasaka

**Affiliations:** Department of Applied Life Science, Sojo University, 4-22-1 Ikeda, Nishiku, Kumamoto 860-0082, Japan; miyasaka@bio.sojo-u.ac.jp

This Special Issue aims to contribute to the current knowledge in the field and promote the practical application of photosynthetic bacteria (PSB) biotechnology. Among various PSB, purple non-sulfur bacteria (PNSB) in particular have a wide range of biotechnological applications in agriculture [1,2], aquaculture [3], biomaterial production [4,5,6], renewable energy production [7,8], wastewater treatment [9], and bioremediation [10].

In this Special Issue, Koga et al. [11] reported the application of marine PNSB as probiotics for shrimp aquaculture. Segura et al. [12] used a mixture of volatile fatty acids (VFAs) as a carbon source for *Rhodospirillum rubrum* and discovered their effects on CO_2_ metabolic pathways and cellular redox balance. Edreira et al. [13] reported the effects of voltage on the performance of bioelectrochemical systems to fix CO_2_ with a mixed culture of PNSB and purple sulfur bacteria (PSB).

The most widely used application of PNSB is to promote plant growth and improve the quality of food crops in agriculture. This Special Issue includes two reviews on PNSB applications in agriculture. Lee et al. [14] provided a comprehensive overview of the beneficial effects of PNSB in agriculture, as well as their accomplishments in developing elite PNSB for agriculture. Maeda [15] gave an in-depth analysis of the mechanisms and regulations of nitrogen fixation by nitrogenase of PNSB, and he also provided field data on PNSB’s contribution to nitrogen fixation in rice fields.

Hayashi et al. [16] reported the growth-promoting effect of lipopolysaccharide (LPS) from PNSB in plants for the first time, and the effective concentration of LPS from *Rhodobacter sphaeroides* was 10 pg/mL. In mammals, LPS acts as an endotoxin through the Toll-like receptor 4 (TLR4) signaling pathway at a concentration of pg/mL to ng/mL, causing inflammatory responses. In plants, LPS also acts as an inducer of immune response, but the effective concentration of LPS from various Gram-negative bacteria in plants reportedly ranged from 10 μg to 100 μg/mL [17,18]. The effective concentration of *R. sphaeroides* LPS for the plant was therefore approximately millions of times lower than those reported in previous studies. Iwai et al. [19] reported that biopriming by LPS from *R. sphaeroides* at a concentration of 5 ng/mL improved the root growth of rice seedlings, providing further evidence of the effectiveness of LPS from PNSB in plants at low concentrations. The much lower effective concentration of PNSB LPS than those of other bacteria is likely attributable to its unique lipid A property. Lipid A, a domain of LPS, is a hydrophobic molecule that anchors LPS to the outer membrane of Gram-negative bacteria [20]. Lipid A acts as the active component of the endotoxicity of LPS. The LPS of *R. sphaeroides* and its lipid A are known to exhibit unique properties in mammals [21]. They show no endotoxic activity but have an endotoxin-antagonistic activity for TLR4. The chemically synthesized lipid A of *R. sphaeroides*, named eritoran (E5564; Eisai), has also been developed for therapeutic application [22], and eritoran has been shown to protect animals from inflammation by blocking the TLR4 signaling pathway [23,24]. In view of these facts, it will be of great interest to investigate the effects of lipid A from *R. sphaeroides*, as well as eritoran, on plant growth in the future.
